# Supplementary material for: A chromosome-level genome assembly for the amphibious plant Rorippa aquatica reveals its allotetraploid origin and mechanisms of heterophylly upon submergence
Source: Commun Biol. 2024 Apr 18;7:431. doi: 10.1038/s42003-024-06088-7 (PMC11026429; doi:10.1038/s42003-024-06088-7)
Supplement: Supplementary file 2 — Supplementary Information [file 42003_2024_6088_MOESM2_ESM.pdf]

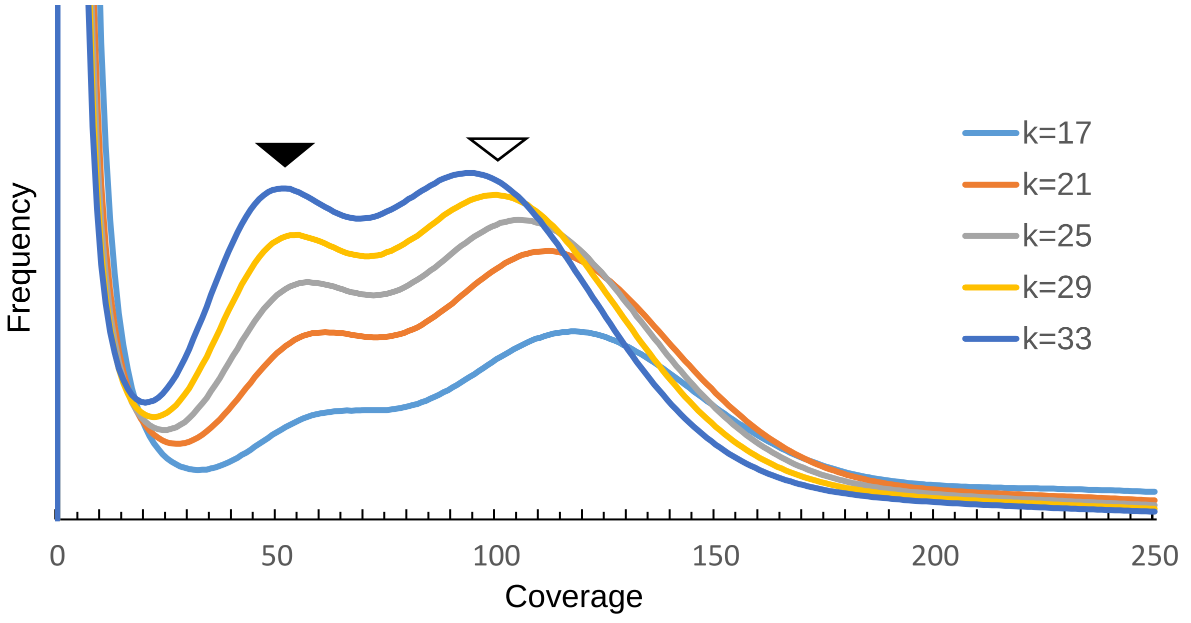

Supplementary Figure 1. K-mer distribution of genome-seq reads of *R. aquatica*. Solid and open arrowhead indicates K-mer peak from heterozygous and homozygous region, respectively.

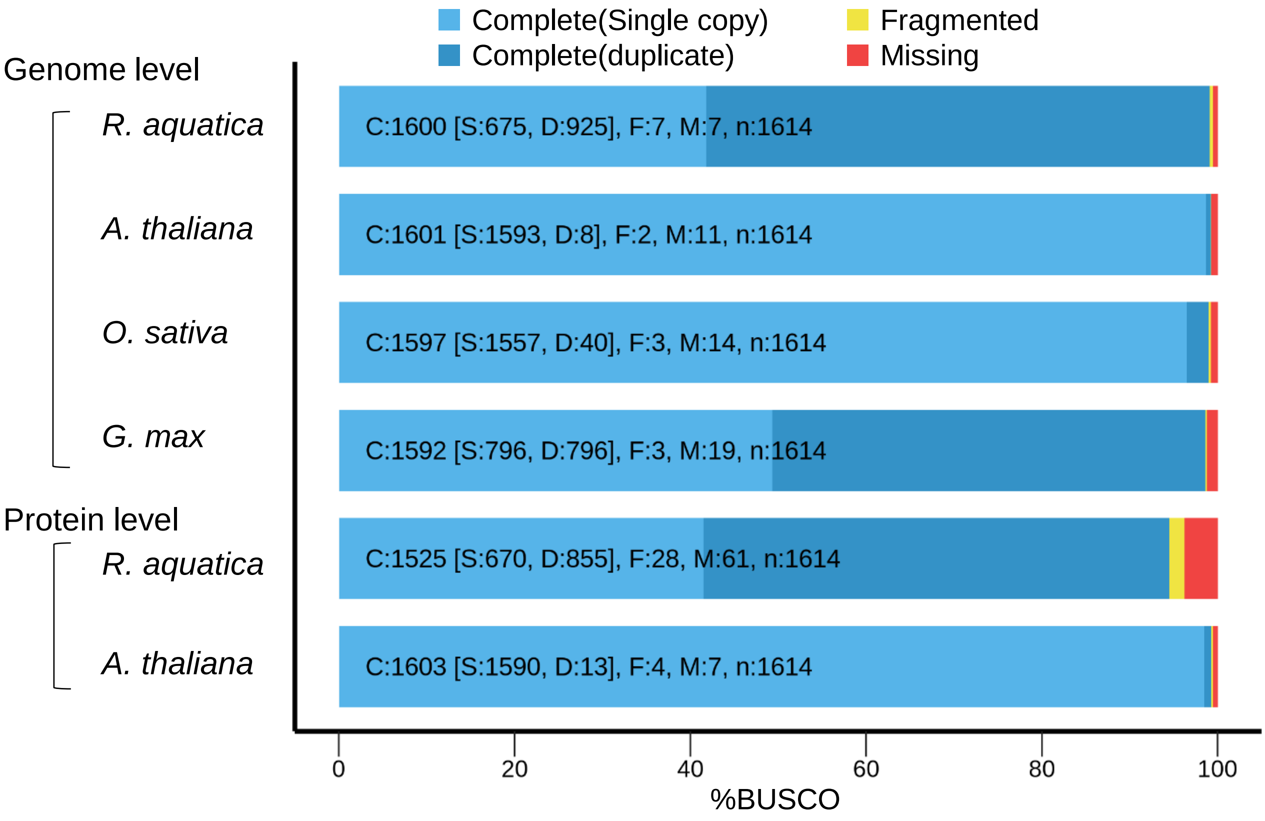

Supplementary Figure 2. Quality assessment of *R. aquatica* genome by BUSCO.

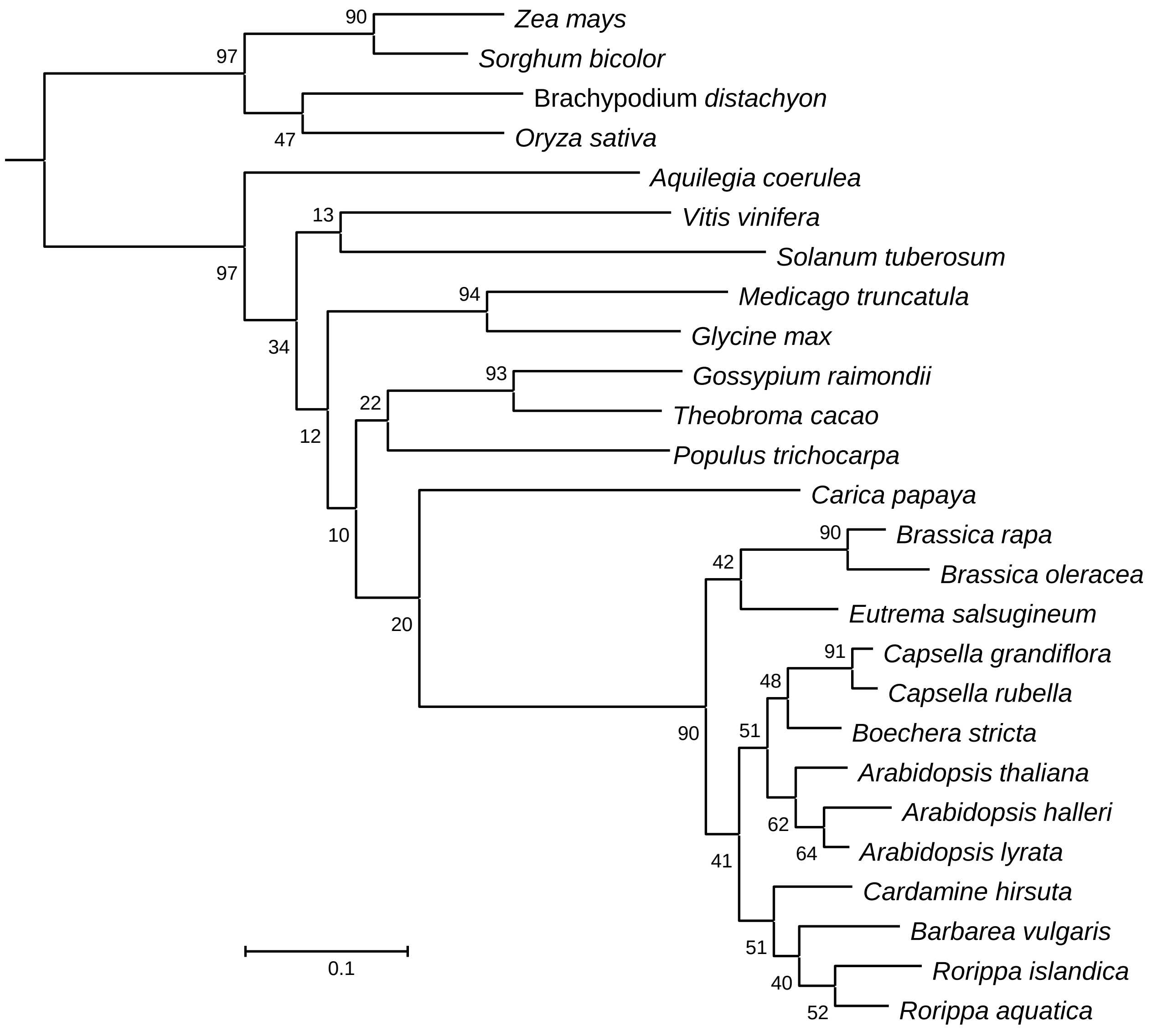

Supplementary Figure 3. Phylogenetic tree from whole-genome level data. Scale bar indicates branch length (substitutions per site).

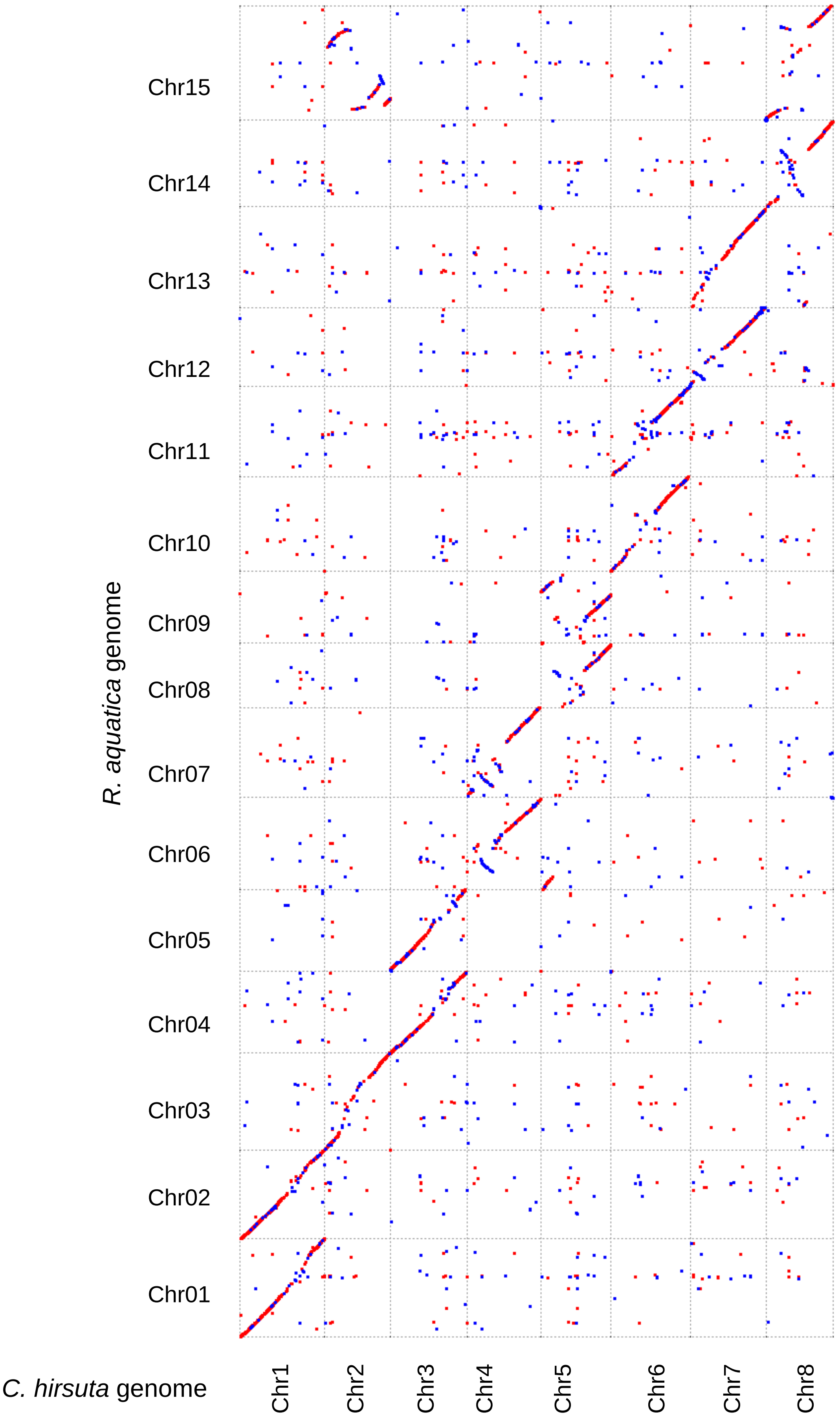

Supplementary Figure 4. Comparison of genome sequences between *Cardamine hirsuta* and *Rorippa aquatica*. Dots with red and blue indicate the region that show similarity to forward and reverse strand of *C. hirsuta* genome respectively.

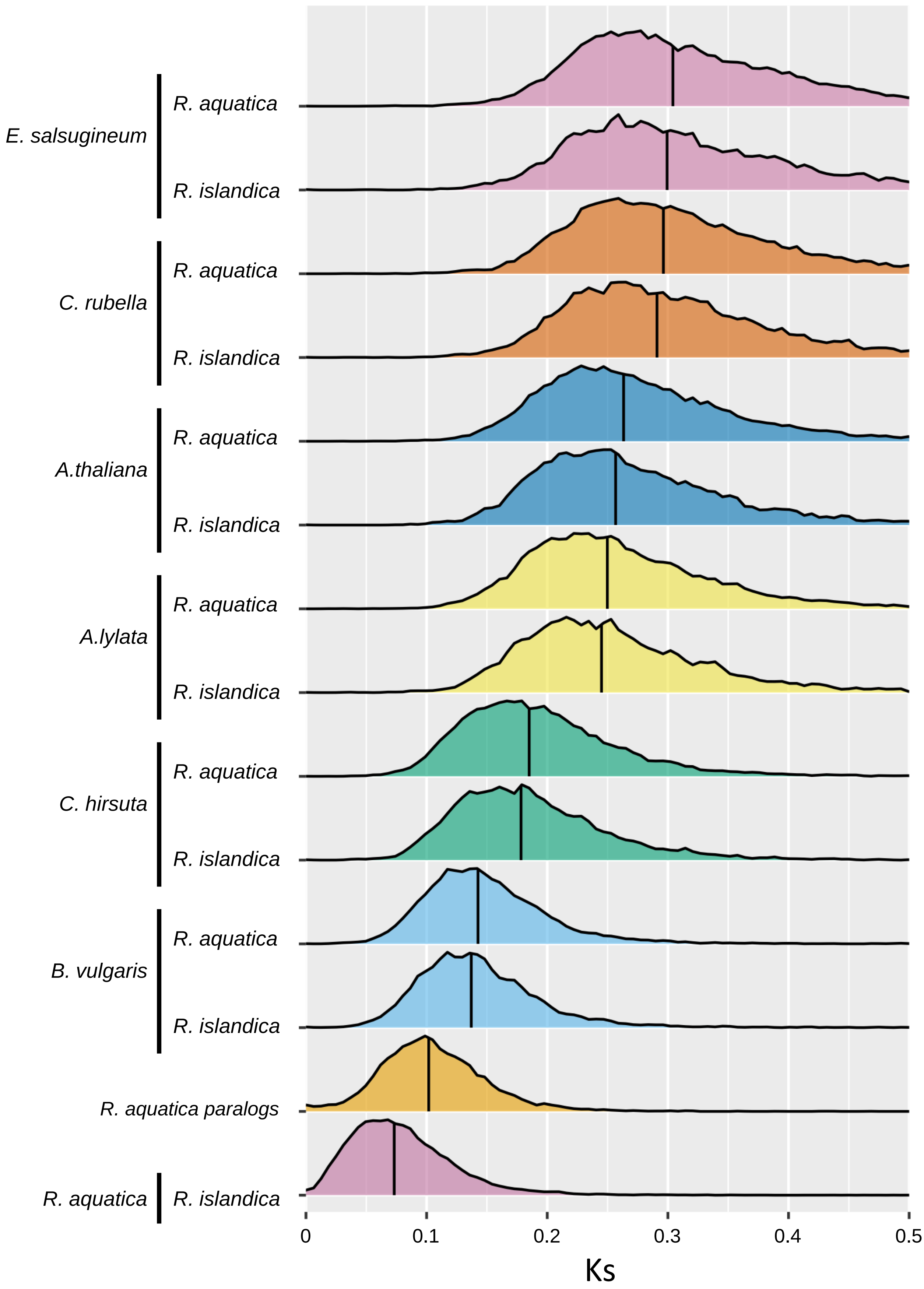

Supplementary Figure 5. Ks distribution among genes that are conserved as single copy in diploid Brassicaceae species but duplicated in *R. aquatica*. Vertical lines on plot show median.

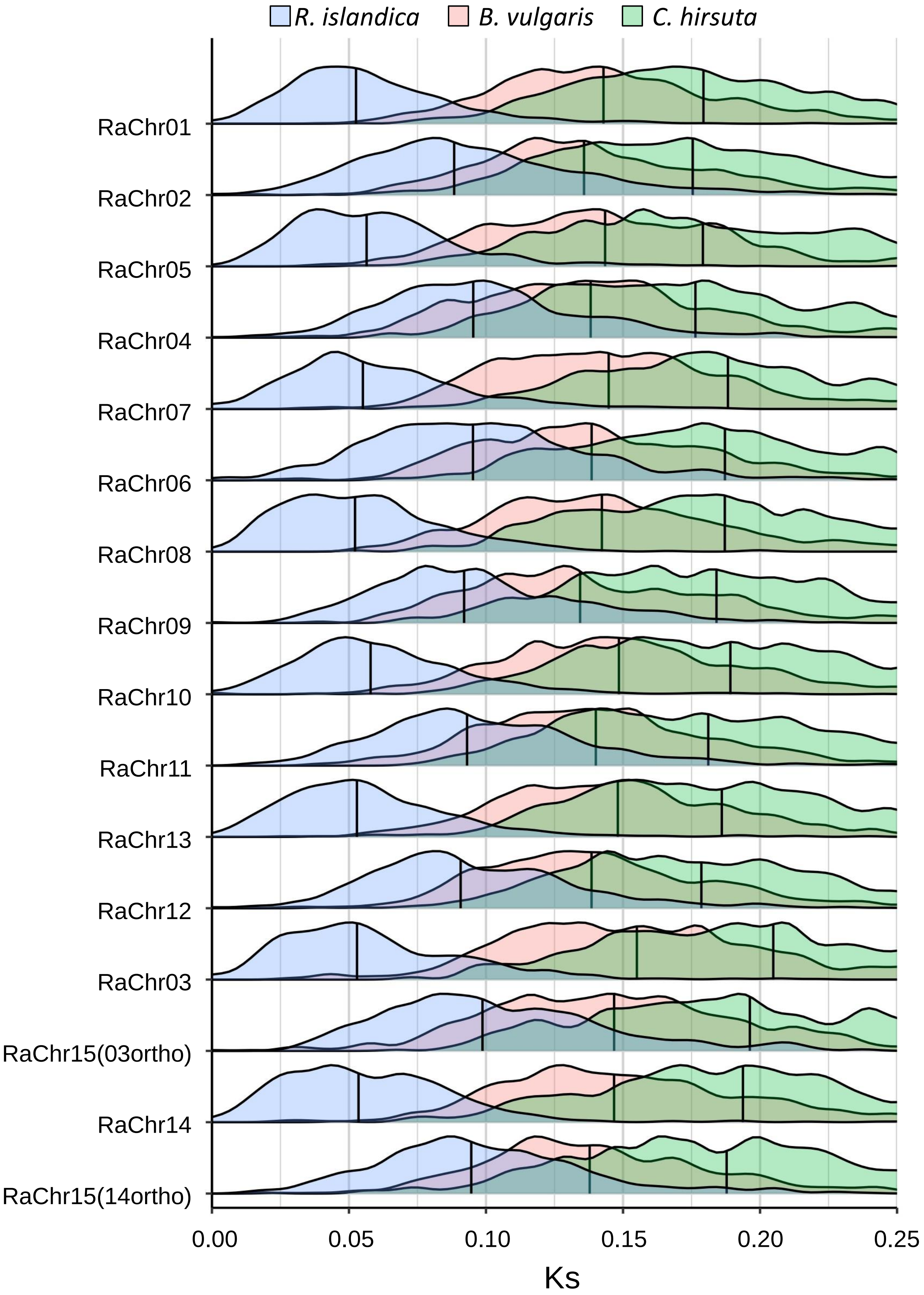

Supplementary Figure 6. Chromosome level Ks distribution between *R. aquatica* and 3 Brassicaceae species (*R. islandica*, *B. vulgaris* and *C. hirsuta*). Vertical lines on plot show median.

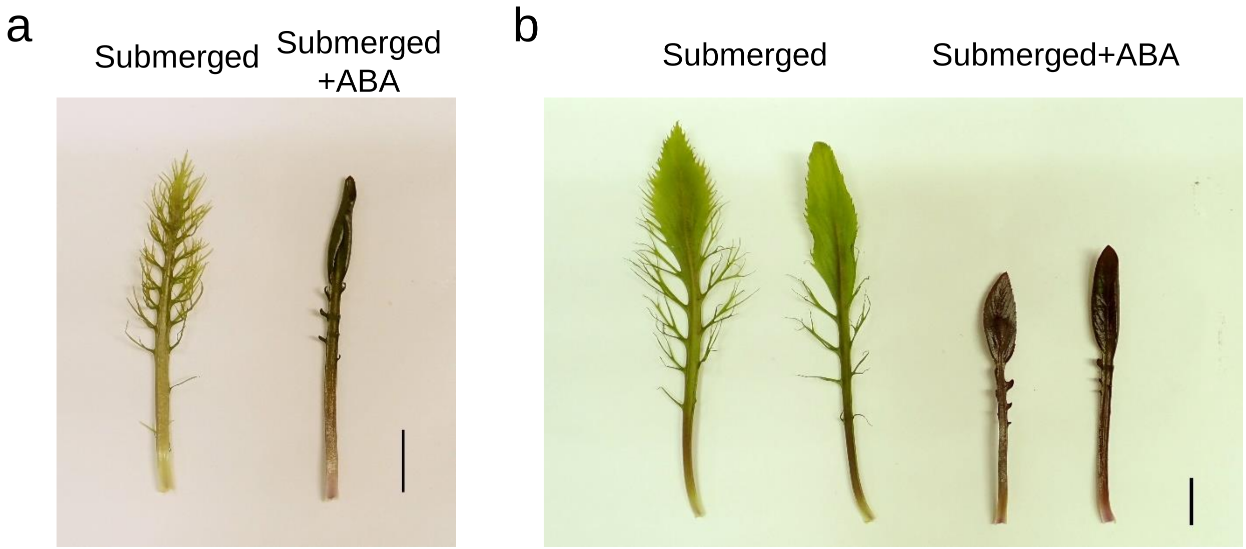

Supplementary Figure 7. Effect of abscisic acid on *R. aquatica* heterophylly in response to submergence. (a) Young leaves (2nd leaf) under control submerged condition and exogenous ABA treatment. (b) Expanded leaves (3rd and 4th leaves) under control submergence and exogenous ABA treatment. Plants were transferred from terrestrial to submerged condition at 25 ˚ C without or with 10 μM ABA and grown for 3 weeks. Leaf number was defined youngest leaf (leaf length > 10 mm) as 1st leaf. Scale bars indicate 10mm.

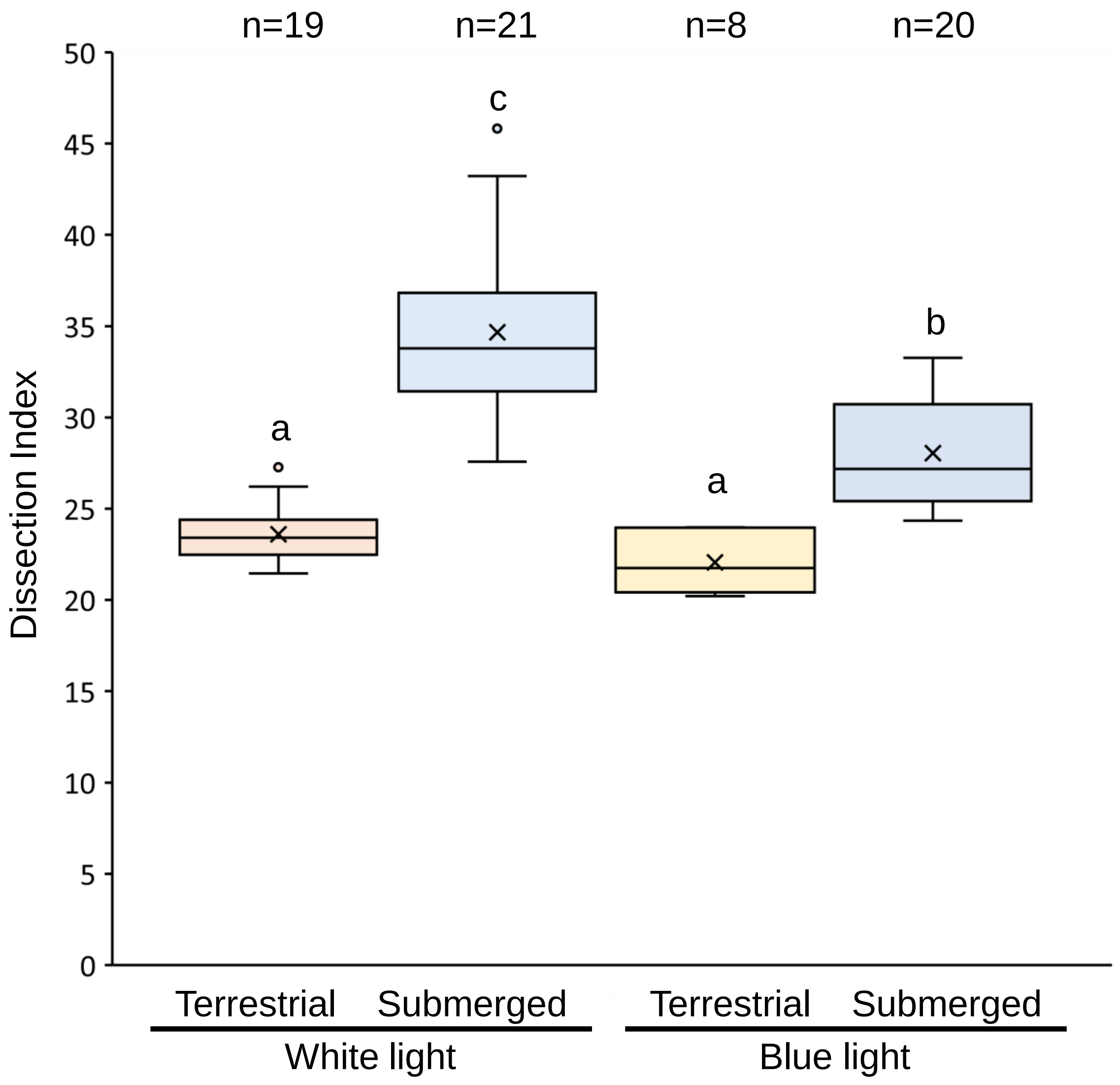

Supplementary Figure 8. Statistical analysis of leaf complexity. Box plot shows Dissection Index calculated by perimeter / (square root of area). The crosses and central horizontal bars indicate mean and median, respectively. The lower and upper limits of the boxes are the first and third quartiles, respectively. Letters near plots indicates statistic group based on adjusted p-value ($p < 0.05$) with Tukey-Kramer test.

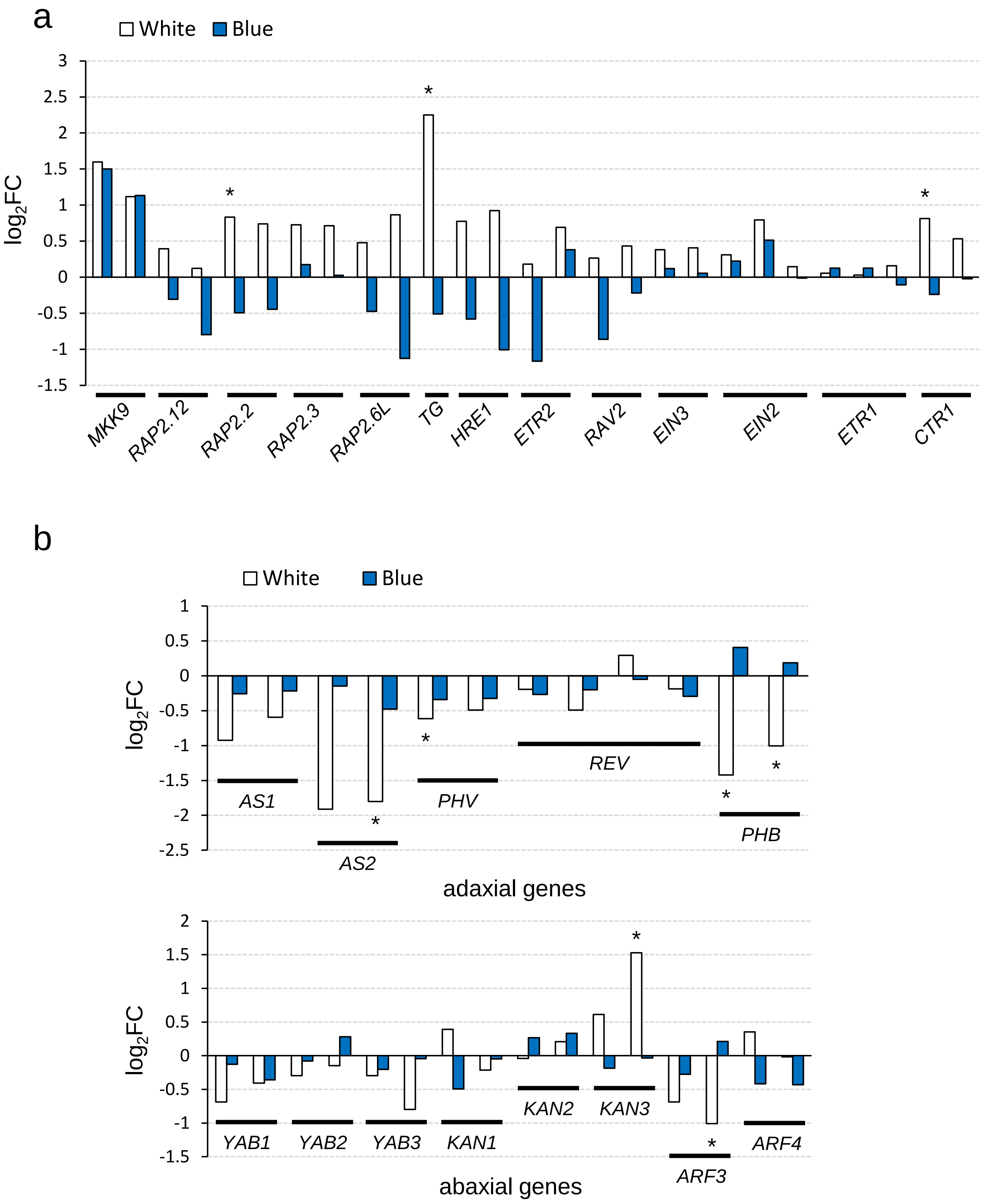

Supplementary Figure 9. Expression profile of ethylene response genes (a) and adaxial-abaxial polarity genes (b) after transfer to submergence under white or blue light condition. Graphs depict fold change at 1 hour after transfer from terrestrial condition under white light to submerged condition under white or blue light. "*" indicates significant difference (FDR < 0.01) against reference sample (Terrestrial under white light condition).

Supplementary Table 1. Summary of assembled *R. aquatica* genome sequences.

Number of Chromosomal sequences	15
Total number of sequences	2,055
Num. of sequences (>= 50kbp)	120
Total length（bp）	452,230,509
Total length (>= 50kbp)	414,395,297
Total length (Chromosomes)	414,395,297
Length of longest sequence	35,558,744
GC (%)	35.31
N50	28,163,506

Supplementary Table 2. Summary of genome size estimation by k-mer counting.

	k=17	k=21	k=25	k=29	k=33
total number of k-mers ($x10^6$)	49,594	47,728	45,964	44,055	42,019
peak of k-mer distribution	117	111	105	100	95
estimated genome size (Mb)	423.89	429.99	437.76	440.55	442.31

Supplementary Table 3. Statistics of repeat masking.

	Number of element	Length	% of genome
SINEs	1,801	354,676	0.08
LINEs	9,692	9,767,096	2.16
LTR_elements	120,041	133,198,170	29.45
DNA_elements	57,852	22,871,963	5.06
Unclassified	85,028	42,031,712	9.29
total	274,414	208,223,617	46.04

Supplementary Table 4. Statistics of predicted gene structure.

	transcript	gene
number	94666	46197
max length	16567	16567
minimum length	150	150
average length	1912.4	1458.7
median length	1775	1266

Supplementary Table 5. Gene distribution in 2 *Rorippa* species classified into Brassicaceae conserved single gene orthogroup.

Gene number in orthogroup	*R. aquatica*		*R. islandica*	
	number	ratio(%)	number	ratio(%)
0	517	4.8	453	4.2
1	3695	34.0	9939	91.5
2	6373	58.6	380	3.5
3	260	2.4	76	0.7
4	18	0.2	15	0.1
>=5	5	0.0	5	0.0
Total	10868	100.0	10868	100.0

Supplementary Table 6. Divergence time estimated from Ks. Age estimates given as million years ago.

	R. aquatica				*R. islandica*			
	Ks		age		Ks		age	
	average	median	average	median	average	median	average	median
E. salsugineum	0.331	0.304	25.4	23.3	0.326	0.299	25.0	22.9
C. rubella	0.316	0.296	24.3	22.7	0.311	0.291	23.8	22.3
A. thaliana	0.281	0.264	21.5	20.2	0.275	0.257	21.1	19.7
A. lyrata	0.266	0.250	20.4	19.2	0.260	0.245	20.0	18.8
C. hirsuta	0.196	0.185	15.0	14.2	0.189	0.178	14.5	13.7
B. vulgaris	0.151	0.142	11.6	10.9	0.146	0.137	11.2	10.5
R. islandica	0.081	0.073	6.2	5.6				
R. aquatica paralogs	0.108	0.102	8.3	7.8				

Supplementary Table 7. Median Ks values at chromosome level between *R. aquatica* and *R. islandica*. Letters at median show groups assigned based on fold discovery rate (FDR) by pairwise wilcoxon rank sum tests (FDR < 0.05)

Subgenome A group		Subgenome B group	
RaChr01	0.0526 [ab]	RaChr02	0.0884 [d]
RaChr05	0.0564 [bc]	RaChr04	0.0954 [ef]
RaChr07	0.0551 [bc]	RaChr06	0.0953 [ef]
RaChr08	0.0522 [a]	RaChr09	0.0920 [de]
RaChr10	0.0579 [c]	RaChr11	0.0931 [de]
RaChr13	0.0529 [ab]	RaChr12	0.0908 [de]
RaChr03	0.0529 [ab]	RaChr15(03ortho)	0.0987 [f]
RaChr14	0.0535 [ab]	RaChr15(14ortho)	0.0946 [ef]
Total	0.0541	Total	0.0935

Supplementary Table 8. Statistics analysis of Ks between *R. aquatica* chromosome and *R. islandica* by pairwise wilcoxon rank sum tests. Values show FDR. Pale blue and orange indicates significant difference with FDR < 0.05 and <2e-16, respectively.

	Subgenome A								Subgenome B						
	RaChr01	RaChr03	RaChr05	RaChr07	RaChr08	RaChr10	RaChr13	RaChr14	RaChr02	RaChr04	RaChr06	RaChr09	RaChr11	RaChr12	RaChr15 (03ortho)
**RaChr01**	-	-	-	-	-	-	-	-	-	-	-	-	-	-	-
**RaChr03**	0.80385	-	-	-	-	-	-	-	-	-	-	-	-	-	-
**RaChr05**	0.16474	0.37411	-	-	-	-	-	-	-	-	-	-	-	-	-
**RaChr07**	0.10002	0.28505	0.72836	-	-	-	-	-	-	-	-	-	-	-	-
**RaChr08**	0.29485	0.26331	0.02451	0.01562	-	-	-	-	-	-	-	-	-	-	-
**RaChr10**	0.00155	0.02895	0.1434	0.34319	0.00029	-	-	-	-	-	-	-	-	-	-
**RaChr13**	0.89277	0.72836	0.14954	0.10049	0.39479	0.00253	-	-	-	-	-	-	-	-	-
**RaChr14**	0.92087	0.80385	0.30465	0.18251	0.3077	0.01454	0.83646	-	-	-	-	-	-	-	-
**RaChr02**	< 2e-16	< 2e-16	< 2e-16	< 2e-16	< 2e-16	< 2e-16	< 2e-16	< 2e-16	-	-	-	-	-	-	-
**RaChr04**	< 2e-16	< 2e-16	< 2e-16	< 2e-16	< 2e-16	< 2e-16	< 2e-16	< 2e-16	0.00826	-	-	-	-	-	-
**RaChr06**	< 2e-16	< 2e-16	< 2e-16	< 2e-16	< 2e-16	< 2e-16	< 2e-16	< 2e-16	0.11941	0.5326	-	-	-	-	-
**RaChr09**	< 2e-16	< 2e-16	< 2e-16	< 2e-16	< 2e-16	< 2e-16	< 2e-16	< 2e-16	0.24147	0.29485	0.76432	-	-	-	-
**RaChr11**	< 2e-16	< 2e-16	< 2e-16	< 2e-16	< 2e-16	< 2e-16	< 2e-16	< 2e-16	0.02478	0.76158	0.74188	0.49727	-	-	-
**RaChr12**	< 2e-16	< 2e-16	< 2e-16	< 2e-16	< 2e-16	< 2e-16	< 2e-16	< 2e-16	0.26473	0.17016	0.62886	0.83086	0.29485	-	-
**RaChr15(03ortho)**	< 2e-16	< 2e-16	< 2e-16	< 2e-16	< 2e-16	< 2e-16	< 2e-16	< 2e-16	0.000058	0.138	0.0383	0.02014	0.06322	0.00393	-
**RaChr15(14ortho)**	< 2e-16	< 2e-16	< 2e-16	< 2e-16	< 2e-16	< 2e-16	< 2e-16	< 2e-16	0.02091	0.91637	0.62916	0.37411	0.83086	0.234	0.1282
