# Supplementary material for: A chromosome-level genome assembly for the amphibious plant Rorippa aquatica reveals its allotetraploid origin and mechanisms of heterophylly upon submergence
Source: Commun Biol. 2024 Apr 18;7:431. doi: 10.1038/s42003-024-06088-7 (PMC11026429; doi:10.1038/s42003-024-06088-7)
Supplement: Supplementary file 3 — Description of Additional Supplementary Files [file 42003_2024_6088_MOESM3_ESM.pdf]

Description of Additional Supplementary Files

File name: Supplementary Data 1

Description: Ortholog clustering in Rorippa and Brassicaceae.

File name: Supplementary Data 2

Description: RNA-seq Expression profile data.

File name: Supplementary Data 3

Description: GO enrichment analysis for DEG at submergence.

File name: Supplementary Data 4

Description: Common DEGs inducing submerged-type leaf.

File name: Supplementary Data 5

Description: Information of RNAseq read and mapping statistics

File name: Supplementary Data 6

Description: Numerical source data for graphs
