# Supplementary material for: A chromosome-level genome assembly for the amphibious plant Rorippa aquatica reveals its allotetraploid origin and mechanisms of heterophylly upon submergence
Source: Commun Biol. 2024 Apr 18;7:431. doi: 10.1038/s42003-024-06088-7 (PMC11026429; doi:10.1038/s42003-024-06088-7)
Supplement: Supplementary file 10 — Reporting Summary [file 42003_2024_6088_MOESM10_ESM.pdf]

nature portfolio

Corresponding author(s): Seisuke Kimura

Last updated by author(s): Mar 18, 2024

Reporting Summary

Nature Portfolio wishes to improve the reproducibility of the work that we publish. This form provides structure for consistency and transparency in reporting. For further information on Nature Portfolio policies, see our Editorial Policies and the Editorial Policy Checklist.

Statistics

For all statistical analyses, confirm that the following items are present in the figure legend, table legend, main text, or Methods section.

n/a	Confirmed	
☐	☒	The exact sample size (*n*) for each experimental group/condition, given as a discrete number and unit of measurement
☐	☒	A statement on whether measurements were taken from distinct samples or whether the same sample was measured repeatedly
☐	☒	The statistical test(s) used AND whether they are one- or two-sided<br>*Only common tests should be described solely by name; describe more complex techniques in the Methods section.*
☒	☐	A description of all covariates tested
☐	☒	A description of any assumptions or corrections, such as tests of normality and adjustment for multiple comparisons
☐	☒	A full description of the statistical parameters including central tendency (e.g. means) or other basic estimates (e.g. regression coefficient) AND variation (e.g. standard deviation) or associated estimates of uncertainty (e.g. confidence intervals)
☐	☒	For null hypothesis testing, the test statistic (e.g. *F*, *t*, *r*) with confidence intervals, effect sizes, degrees of freedom and *P* value noted<br>*Give P values as exact values whenever suitable.*
☒	☐	For Bayesian analysis, information on the choice of priors and Markov chain Monte Carlo settings
☒	☐	For hierarchical and complex designs, identification of the appropriate level for tests and full reporting of outcomes
☐	☒	Estimates of effect sizes (e.g. Cohen's *d*, Pearson's *r*), indicating how they were calculated

Our web collection on statistics for biologists contains articles on many of the points above.

Software and code

Policy information about availability of computer code

Data collection	No software was used.
Data analysis	jellyfish2, MaSuRCA 3.2.8, Pilon, 3D de novo assembly (3D-DNA) pipeline v180922, LR_Gapcloser, ColorMap, BUSCO v4, RepeatModeler, RepeatMasker, PASA pipeline v2.3.3, AUGUSTUS 3.3.2, GlimmerHMM 3.0.2, exonerate 2.2.0, EvidenceModeler (EVM) 1.1.1, Blast2GO, MUMMER 3.23, circos 0.69, OrthoFinder 2.27, MACSE v2.03, PAML 4.8, Tophat2, edgeR, ImageJ 1.54d

For manuscripts utilizing custom algorithms or software that are central to the research but not yet described in published literature, software must be made available to editors and reviewers. We strongly encourage code deposition in a community repository (e.g. GitHub). See the Nature Portfolio guidelines for submitting code & software for further information.

Data

Policy information about availability of data

All manuscripts must include a data availability statement. This statement should provide the following information, where applicable:

- Accession codes, unique identifiers, or web links for publicly available datasets
- A description of any restrictions on data availability
- For clinical datasets or third party data, please ensure that the statement adheres to our policy

The assembled R. aquatica genome sequences and their annotations were deposited in Figshare (10.6084/m9.figshare.19207362). The draft assembly data of R. islandica were deposited in Figshare (10.6084/m9.figshare.24849201). Genome-seq read data and Hi-C seq read data are available in the DDBJ Sequenced Read

Archive (DRA) under the accession numbers DRA010675 and DRA013596, respectively. Transcriptome read data are also available in DRA under DRA014113, DRA014114, DRA014164, and DRA014165. All other data are available from the corresponding author on reasonable request.

Research involving human participants, their data, or biological material

Policy information about studies with human participants or human data. See also policy information about sex, gender (identity/presentation), and sexual orientation and race, ethnicity and racism.

Reporting on sex and gender	not applicable
Reporting on race, ethnicity, or other socially relevant groupings	not applicable
Population characteristics	not applicable
Recruitment	not applicable
Ethics oversight	not applicable

Note that full information on the approval of the study protocol must also be provided in the manuscript.

Field-specific reporting

Please select the one below that is the best fit for your research. If you are not sure, read the appropriate sections before making your selection.

☒ Life sciences ☐ Behavioural & social sciences ☐ Ecological, evolutionary & environmental sciences

For a reference copy of the document with all sections, see nature.com/documents/nr-reporting-summary-flat.pdf

Life sciences study design

All studies must disclose on these points even when the disclosure is negative.

Sample size	No statistical methods were used to pre-determine sample sizes but our sample sizes are similar to those reported in previous publications.
Data exclusions	No data were excluded from the analyses.
Replication	All experiments were repeated at least 3 times to verify the reproducibility.
Randomization	For morphological and RNA-seq analyses, samples were randomly allocated to each experimental conditions from growth condition.
Blinding	Blindings were not performed in the study. No bias to plant samples could be made by investigator due to automatic adjustment of experimental growth conditions in growth chamber.

Reporting for specific materials, systems and methods

We require information from authors about some types of materials, experimental systems and methods used in many studies. Here, indicate whether each material, system or method listed is relevant to your study. If you are not sure if a list item applies to your research, read the appropriate section before selecting a response.

Materials & experimental systems

n/a	Involved in the study	
☒	☐	Antibodies
☒	☐	Eukaryotic cell lines
☒	☐	Palaeontology and archaeology
☒	☐	Animals and other organisms
☒	☐	Clinical data
☒	☐	Dual use research of concern
☐	☒	Plants

Methods

n/a	Involved in the study	
☒	☐	ChIP-seq
☒	☐	Flow cytometry
☒	☐	MRI-based neuroimaging

Plants

Seed stocks	Rorippa aquatica used in the study are kept in laboratory with clonal regeneration.
Novel plant genotypes	No novel plant genotypes were constructed in the study.
Authentication	No novel plant genotypes were constructed in the study.
